# Invasion of Peripheral Immune Cells into Brain Parenchyma after Cardiac Arrest and Resuscitation

**DOI:** 10.14336/AD.2017.0926

**Published:** 2018-06-01

**Authors:** Can Zhang, Nicole R. Brandon, Kerryann Koper, Pei Tang, Yan Xu, Huanyu Dou

**Affiliations:** ^1^Departments of Anesthesiology; ^2^Pharmacology and Chemical Biology; ^3^Computational and Systems Biology; ^4^Physics and Astronomy, and; ^5^Structural Biology, University of Pittsburgh, Pittsburgh, PA 15213, USA; ^6^Department of Biomedical Sciences, Paul L. Foster School of Medicine, and; ^7^Graduate School of Biomedical Sciences, Texas Tech University Health Sciences Center, El Paso, TX 79905, USA

**Keywords:** immune responses, cerebral global ischemia, CD11b+CD45+, CD8+, blood-brain barrier, MRI inflammation

## Abstract

Although a direct link has long been suspected between systemic immune responses and neuronal injuries after stroke, it is unclear which immune cells play an important role. A question remains as to whether the blood brain barrier (BBB) is transiently disrupted after circulatory arrest to allow peripheral immune cells to enter brain parenchyma. Here, we developed a clinically relevant cardiac arrest and resuscitation model in mice to investigate the BBB integrity using noninvasive magnetic resonance imaging. Changes in immune signals in the brain and periphery were assayed by immunohistochemistry and flow cytometry. Quantitative variance maps from T1-weighted difference images before and after blood-pool contrast clearance revealed BBB disruptions immediately after resuscitation and one day after reperfusion. Time profiles of hippocampal CA1 neuronal injuries correlated with the morphological changes of microglia activation. Cytotoxic T cells, CD11b^+^CD11c^+^ dendritic cells, and CD11b^+^CD45^+hi^ monocytes and macrophages were significantly increased in the brain three days after cardiac arrest and resuscitation, suggesting direct infiltration of these cells following the BBB disruption. Importantly, these immune cell changes were coupled with a parallel increase in the same subset of immune cell populations in the bone marrow and blood. We conclude that neurovascular breakdown during the initial reperfusion phase contributes to the systemic immune cell invasion and subsequent neuropathogenesis affecting the long-term outcome after cardiac arrest and resuscitation.

The 2016 American Heart Association report on heart disease and stroke [1] again showed that of all out-of-hospital cardiac arrest cases assessed by the Emergency Medical Services (EMS), the rate of survival to hospital discharge after cardiac arrest remains extremely low; in 2016 the rate was 6.3%. The survival rate improves to 12% among those treated by EMS, but survival with good neurological function is less than 10% [1]. Among those who are successfully resuscitated, a significant percentage dies subsequently in hospital due to the so-called post-resuscitation syndrome [2].

Despite many decades of investigation offering mechanistic insights into brain injuries and repairs after global ischemia, most of the existing therapeutic approaches, with the possible but inconclusive exception of mild hypothermia [3, 4], have failed to improve outcomes. Although it has long been suspected that the primary event of global ischemia during cardiac arrest triggers a systemic immune reaction leading to a cascade of deteriorating changes in the brain, very little is known about the roles played by the immune cells in neuronal injuries after cardiac arrest and resuscitation. In acute focal ischemia, it is believed that a robust immune response to brain injury can occur even when immune cells are not found in the brain tissues [5, 6]. However, the injury pattern after global ischemia is distinctly different from focal ischemia, and the immune responses under these two conditions should not be assumed to be the same. With the possible breakdown of the blood-brain barrier (BBB) after cardiac arrest and resuscitation, blood components, including erythrocytes, leukocytes, monocytes, and lymphocytes, may gain transient access to the brain parenchyma upon reperfusion. Thus, in addition to the activation of resident immune cells such as microglia, a systemic inflammatory response also likely involves migration and infiltration of peripheral immune cells, setting the stage for both tissue damage and subsequent repair.

In the present study, we developed a clinically relevant cardiac arrest and resuscitation model in mice and investigated the crosstalk between global cerebral ischemia and immunity. Understanding this crosstalk is essential for developing new diagnostic and therapeutic strategies because not only will patterns of peripheral immune cell markers be identified and linked to prognosis of neurological outcomes [7], but also the post-resuscitation immune activities can be programmed and tailored in the future to enhance signals for neuronal survival. By combining flow cytometry and magnetic resonance imaging (MRI), we show here that the BBB is indeed briefly compromised immediately after reperfusion and that an increase in the infiltrating CD11b^+^CD11c^+^ and CD11b^+^CD45^+high^ monocytes and macrophages and CD8^+^ cytotoxic T-cells in the brain parenchyma is associated with the neuronal loss and microglia activation. We conclude that neurovascular breakdown during the initial phase of reperfusion contributes to the systemic immune cell invasion and subsequent neuropathogenesis affecting the long-term outcome after cardiac arrest and resuscitation.

## MATERIALS AND METHODS

### A clinically relevant cardiac arrest model in mice

All animal protocols were approved by the Institutional Animal Care and Use Committee of the University of Pittsburgh. Male CD-1 mice, 9.5 ± 1.7 weeks old and weighing 33.1 ± 4.1 g, were used. The animals were housed in a temperature- and humidity-controlled facility with a 12-h light-dark cycle and fed with standard chow for rodents. A total of 29 mice were randomly assigned to naïve (n = 8), sham-operated (n = 6), and cardiac arrest (n = 15) groups. Cardiac arrest animals were under intensive cares immediately after resuscitation and closely monitored in post-resuscitation days.

The cardiac arrest and resuscitation procedure was adapted from a clinically relevant rat model [8-10] with several important modifications. Briefly, after induction with 4% isoflurane, mice were transorally intubated and mechanically ventilated with a mixture of 50% O_2_ and 50% air. In pilot experiments, optimal tidal volume (typically 10.6 ml/kg) and ventilation speed (typically 130 strokes/min) were determined to ensure that blood gases were within the normal physiological range before cardiac arrest. General anesthesia was maintained with 2% isoflurane. Typically, the left femoral artery and vein were cannulated using biocompatible RenaPulse and Renathane tubing (Braintree Scientific, Inc., Braintree, MA), respectively. To obtain oxygenated blood for later resuscitation use, 200 µL of arterial blood were drawn and mixed with 20 µL of a concentrated stock resuscitation solution containing 0.4 mg/ml epinephrine, 0.5 mEq/ml sodium bicarbonate, and 50 U/ml heparin in sterile saline. The arterial blood pressure (ABP) and electrocardiogram (ECG) were continuously recorded using LabChart (ADInstruments Inc., Colorado Springs, CO). To minimize the surgical trauma to the animals, only one arterial line was established. The same line was branched for both ABP monitoring and for blood infusion during resuscitation. Temperature probes were inserted into the right ear and rectum to measure the head and body temperature, respectively. The body temperature was maintained at 36.5 ± 0.7°C using a heating pad and a heating lamp. Precaution was made to avoid heating the tympanic temperature above 37°C, especially during the no-flow period. After the body temperature reached the normothermic range and 5 min before the planned cardiac arrest, the short-acting muscle relaxant vecuronium (0.5 mg/kg) was given subcutaneously to ensure paralysis. Cardiac arrest was induced by an 80-µl bolus injection of esmolol (30 mg/ml), a short-acting β-adrenergic receptor blocker, through the femoral vein, followed by cessation of mechanical ventilation. Electromechanical dissociation typically occurs within 10 s of esmolol injection, leading to circulatory arrest. Resuscitation started 5 min later with 100% O_2_ ventilation and a slow but steady infusion of the oxygenated blood containing the resuscitation mixture. This procedure mimics clinical cardio-pulmonary support, and no chest compressions were needed. The restoration of spontaneous circulation (ROSC) was assessed by reappearance of electrical activity on the ECG monitor and observation of a rapid and spontaneous elevation of ABP after blood infusion was stopped. The animals that failed to achieve ROSC after being infused with all 220-µL of oxygenated blood with resuscitation mixture were excluded from the study. After cannulas were removed and the wound was closed, animals were continuously ventilated with mixtures of O_2_ and air. The percentage of air was gradually increased until spontaneous breathing was fully established, at which point the animals were extubated and returned to their home cages for observation.

**Table 1:** Antibodies Used in Flow Cytometry.

Antigen	Host/Reactivity	Dye	Supplier
Lymphocytic Markers
CD3e	Syrian Hamster/Mouse	V450	BD Biosciences
CD4	Rat/Mouse	PerCP-Cy5.5	BD Biosciences
CD8a	Rat/Mouse	BUV395	BD Biosciences
CD25	Rat/Mouse	PE-Cy7	BD Biosciences
CD45	Rat/Mouse	APC-Cy7	BD Biosciences
FoxP3	Rat/Mouse	Alexa Fluor647	BD Biosciences
CD28	Syrian Hamster/Mouse	PE	BD Biosciences
INFγ	Rat/Mouse	FITC	Abcam
Phagocytic Markers
CD11b	Rat/Mouse	BUV395	BD Biosciences
CD11c	Armenian Hamster/Mouse	APC	eBiosciences
CD45	Rat/Mouse	APC-Cy7	BD Biosciences
CD80	Armenian Hamster/Mouse	FITC	BD Biosciences
CD86	Rat/Mouse	V450	BD Biosciences
F4/80	Rat/Mouse	PE	BD Biosciences
Ly-6C	Rat/Mouse	PerCP-Cy5.5	BD Biosciences
Ly-6G	Rat/Mouse	BV711	BD Biosciences

### Flow cytometry

Three or 10 days after cardiac arrest and resuscitation, selected animals, along with naïve and time-matched sham controls, were re-anesthetized and their blood was collected by cardiac puncture. Immediately after the blood sample collection, the animals were perfused with 0.9% saline, and portions of the brain (cerebrum region) and bone marrows were collected. To purify leukocytes, the blood was treated with a red blood cell lysis buffer, mechanically dissociated, filtered through a 40-µm sterile cell strainer, and further diluted into Hanks balanced salt solution (HBSS). Similarly, bone marrow was mechanically dissociated and passed through a 40-µm sterile cell strainer into a suspension of single cells. Brain tissues were first lysed by tryspin and then passed through a 70-µm sterile cell strainer to obtain a single-cell suspension. Leukocyte separation and cell isolations were achieved by percoll density gradient centrifugation. The blood was layered on a 34-46% percoll density gradient [11, 12]. The single cell suspensions from the brain were re-suspended in 3 mL of 30% percoll underlain with 70% percoll. The percoll gradients were centrifuged at 400×g for 20 min at 4°C. Proper layers of cells were collected and washed with HBSS. Lymphocytic and phagocytic cell markers (Table 1) were labeled by fluorescent antibodies for flow cytometry analyses.

### MRI measurements

A subset of 11 mice was selected to receive MRI scans in the days preceding cardiac arrest (Day -1), and several hours (Day 0) and 1-9 days after cardiac arrest and resuscitation to assess the integrity of the BBB after reperfusion. Repeated imaging was distributed among different mice so that no single mouse contributes to all 11 possible MRI time points. Mice were anesthetized for the MRI scans using ketamine (100 mg/kg) and xylazine (10 mg/kg) intraperitoneal injection. A half-dose boost of anesthetics was administered between scans, midpoint in the MRI session. For T_1_ contrast, 0.3 mmol/kg Gd-DTPA (Magnevist) was given through the tail vein ~9 min before the first T_1_-weighted image. Additional mice without cardiac arrest or contrast agent were scanned to serve as controls.

All MRI scans were performed on a 600-MHz Bruker imaging spectrometer. Body temperature was maintained at 36-37°C by adjusting the temperature set point of the gradient unit and monitored using a rectal probe. Temperature, ECG, and respiration rate were monitored throughout the experiments, and the MRI acquisitions were gated by the respiration signal to remove motion artifacts. T_1_-weighted images were acquired using the multi-slice multi-echo (MSME) sequence with a TE = 12 ms, TR = 500 ms, and NA = 4. T_2_-weighted images were acquired using the rapid acquisition with relaxation enhancement (RARE) sequence, with a TE = 12 ms, TR = 2 s, RARE factor = 8, and NA = 4. Trans-axial sections of T_1_- and T_2_-weighted images were co-registered using Fiji for anatomical reference, with a FOV = 20 mm x 20 mm and a digital resolution of 256 x 128 (zero filled to 256). The slice thickness was 1 mm with an inter-slice space of 0.2 mm for 9 slices. Anatomical regions of interest (ROI) were determined using the co-registered T_2_-weighted images. T_1_-weighted images at 9 and 36 min after the contrast agent were subtracted. The variance maps were generated using the Fiji variance filter with a one-pixel radius. The pixel intensity values of the variance maps (the pixel-by-pixel variances) within the hippocampal ROI were extracted for quantification.

**Figure 1.**
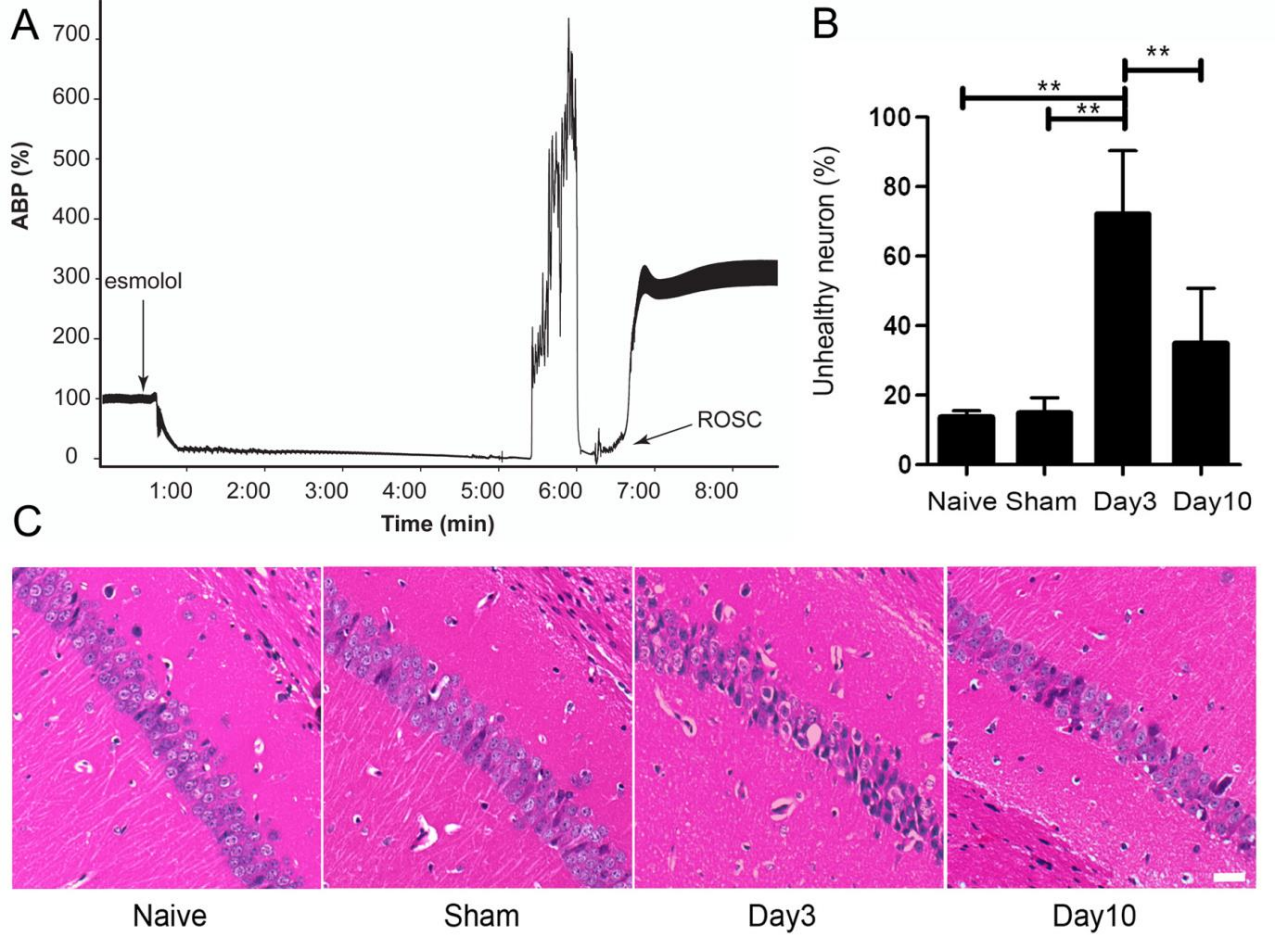
A mouse model of cardiac arrest and resuscitation. (A) Representative trace of relative arterial blood pressure (ABP) before, during, and after a 5-min cardiac arrest and resuscitation. ABP values are normalized against the mean ABP before cardiac arrest to correct for baseline artifacts due to sharing of the same arterial line for blood monitoring and blood infusion during resuscitation. Rapid onset of circulatory arrest was initiated by an intravenous injection of the short-acting-β blocker esmolol, followed by apnea. Controlled resuscitation was achieved by infusion of oxygenated blood with a resuscitation mixture, leading to the return of spontaneous circulation (ROSC) with ~1 min. (B) Quantification of neuronal injuries based on the percentage of unhealthy neurons in the CA1 region of the hippocampus for naïve and sham-operated controls and for cardiac arrest mice 3 and 10 days after cardiac arrest and resuscitation. (C) Representative micrographs of H&E staining in paraffin sections showed that ischemia-damaged hippocampal CA1 pyramidal neurons are significantly increased on post-resuscitation day 3. A decrease in the number of unhealthy neurons is seen on post-resuscitation day 10. Data in (B) are mean ± SEM and analyzed using one-way ANOVA with pair-wise *post hoc* least significant difference comparison. ** *P* < 0.01.

### Immunohistochemistry

To assess histological damage, animals were sacrificed 3 and 10 days after cardiac arrest and resuscitation, and their brains were perfused with saline. Half hemispheres from control and experimental animals were embedded in paraffin, cut into 5 µm coronal sections using a Shandon™ Excelsior Es Processor (Thermo Fisher Scientific, Pittsburgh, PA USA), and then subjected to the standard hematoxylin-eosin (H&E) staining protocol using a Shandon Varistain 24-4 automatic slide stainer. Adjacent tissue sections were subjected to the standard antigen retrieval protocol for immunohistochemistry. After washing and blocking, sections were separately incubated at 4°C overnight with the primary antibodies for glial fibrillary acidic protein (GFAP, Millipore USA, Billerica, MA, 1:250), ionized calcium binding adaptor molecule 1 (*Iba1*, Millipore, 1:200), and CD45 (Abcam, Cambridge, UK, 1:300), followed by washing and 2-h incubation with fluorescent secondary antibodies at room temperature. Nuclear DNA was stained with 4′-6-diamidino-2-phenylindole (DAPI) 1 μg/mL (Sigma-Aldrich Corp., St Louis, MO, USA) in phosphate-buffered saline. After coverslipping with fluoromount-G (Southern Biotech, Birmingham, AL), sections were examined under an Olympus IX-81 fluorescence microscope (Olympus Corp., Tokyo, Japan) controlled by SlideBook software (Intelligent Imaging Innovations, Inc., Denver, CO, USA).

### Statistical Analysis

SPSS software (IBM Corp., Armonk, NY, USA) was used for all statistical analyses. Data are presented as mean ± standard error of the mean (SEM). One-way analysis of variance (ANOVA) with least significant difference (LSD) *post hoc* test was used for multiple comparisons. A *p* value <0.05 is considered statistically significant.

**Figure 2.**
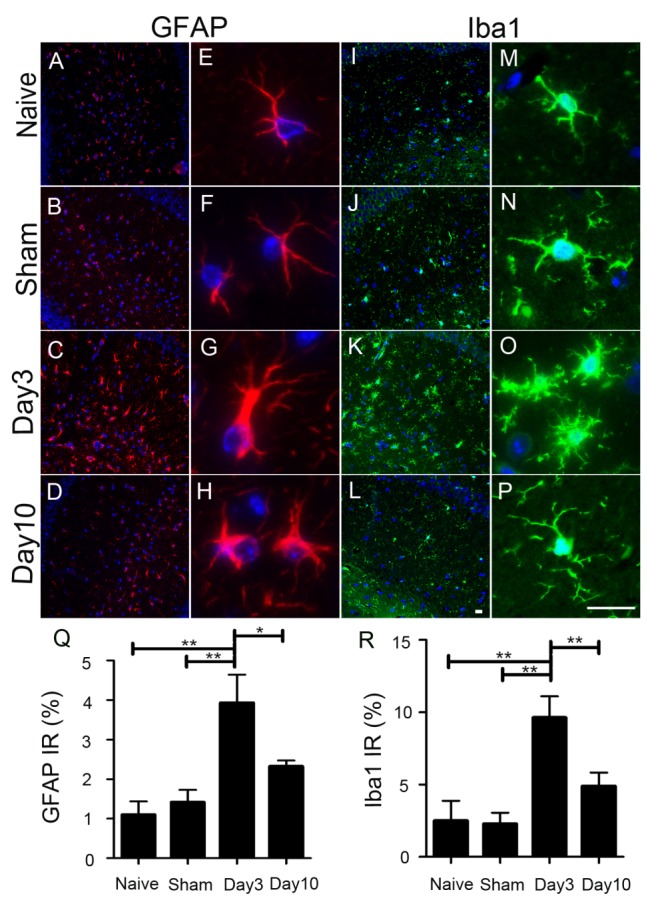
Characterization of astrocytosis and innate immune responses in the central nervous system after cardiac arrest. Displayed here are immune-fluorescent micrographs of DAPI staining (blue) for nuclear DNA, GFAP (red, A-H) for astrocytes, and Iba1 (green, I-P) for reactive microglia, showing the overall immunoreactivity in the hippocampal region (10x, A-D and I-L) and the detailed morphology (40x E-H and M-P) 3 and 10 days after cardiac arrest and resuscitation in comparison to the naïve and time-matched sham controls. Hypertrophy of astrocytic processes (G and H) and amoeboid morphology of macrophagic changes of microglia become very pronounced 3 days after cardiac arrest and resuscitation. The GFAP (Q) and Iba1 (R) immune-reactivities are quantified by fluorescence image segmentation as mean ± SEM. **P < 0.01 and *P < 0.05. (Scale bars = 20 μm)

**Figure 3.**
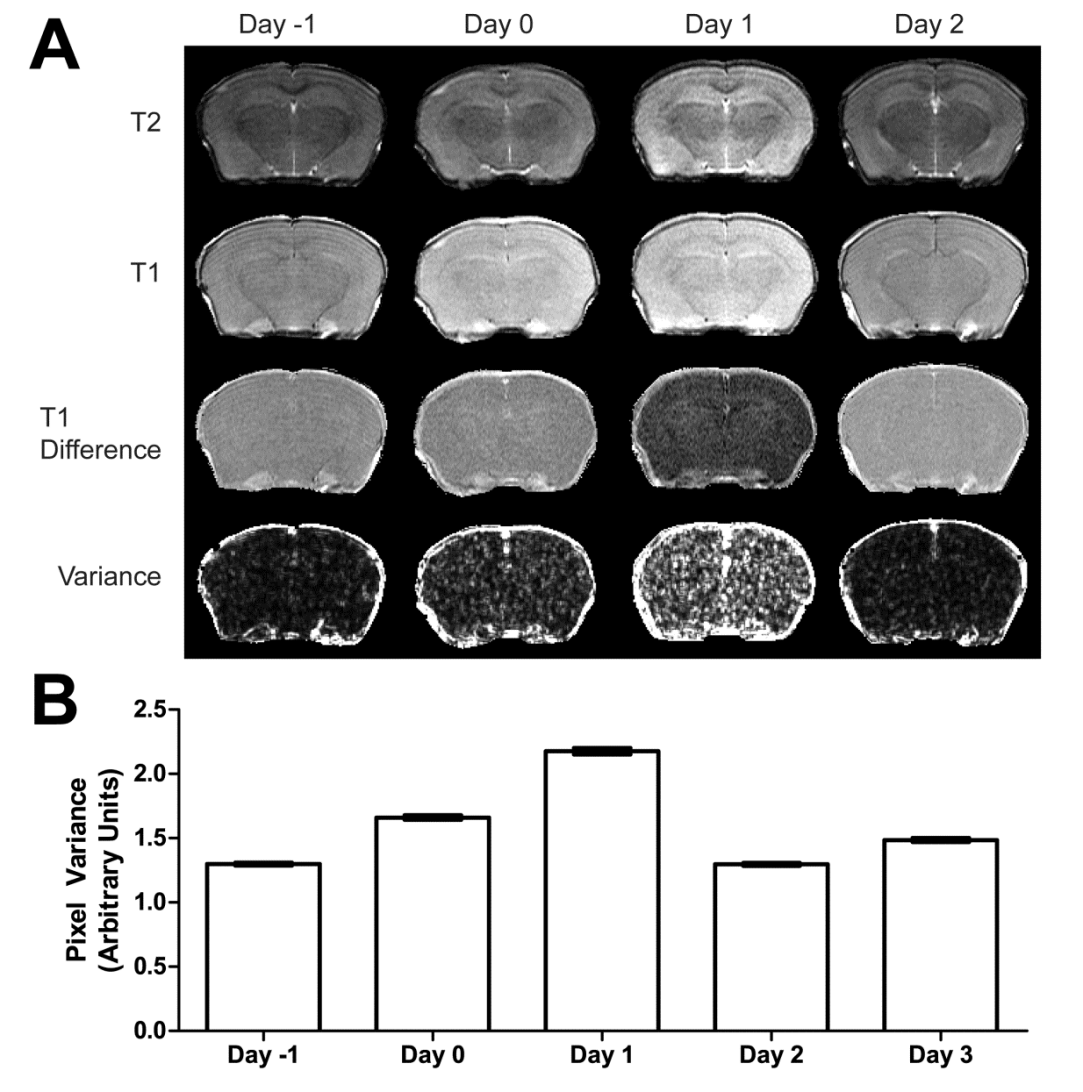
Magnetic resonance imaging (MRI) of blood-brain-barrier (BBB) integrity. (A) Displayed are representative T2- (first row) and T1-weighted (second row) images, T1-difference images (third row), and the corresponding variance maps (fourth row) before cardiac arrest (Day -1), and 0, 1, and 2 days after resuscitation. Images in each row are displayed using the same intensity scales for easy comparison. (B) Pixel variances in the variance maps are averaged (mean ± SEM) to measure the degree of the BBB leakage as a function of time. Nonparametric Kruskal-Wallis test showed that all time points are different from each other with p = 0.000 except Day 0 vs. Day 3 (p = 0.027) and Day 2 vs. Day -1 (not significantly different).

## RESULTS

### Neuronal injury

Our mouse model generates a reproducible circulatory arrest insult. Fig. 1A depicts a representative trace of arterial blood pressure (ABP) of a mouse before, during, and after cardiac arrest and resuscitation. Upon intravenous injection of esmolol, a rapid drop in ABP occurred in 8.7 ± 1.3 s. Resuscitation by infusion of oxygenated blood with the resuscitation mixture began 4.94 ± 0.04 min later, leading to ROSC within 1.1 ± 0.2 min. To minimize surgical trauma to the animals, we use the same arterial line for both arterial blood monitoring and blood infusion. Connecting an infusion pump to the ABP monitor artificially elevated the pressure baseline when the heart was completely stopped. Hence, the ABP in Fig. 1A is displayed not as the absolute values in mmHg, but as percentage changes relative to the mean ABP before cardiac arrest. The peak pressure in Fig. 1A during blood infusion is less than 300 mmHg. To evaluate histological damages, H&E staining was used to quantify the neuronal injuries in the hippocampal CA1 region, where neurons were particularly vulnerable to ischemic insults [8, 13]. Pyramidal neurons with a distinct nucleus are considered healthy, whereas neurons that are hypereosinophilic, or show typical signs of shrinkage and dysmorphic shape, are considered unhealthy [8, 9, 14]. The percentage of unhealthy neurons, counted in the hippocampal CA1 region by five investigators blind of tissue identities, was averaged and quantified for the naïve and sham-operated mice and for mice sacrificed 3 and 10 days after cardiac arrest and resuscitation (Fig. 1B). More than 60% of the CA1 neurons appeared unhealthy when evaluated 3 days after cardiac arrest and resuscitation. The percentage was reduced to ~40% on Day 10. Representative H&E staining micrographs, reflecting the averages of histological changes, are shown in Fig. 1C. We also evaluated the neurologic outcome after cardiac arrest and resuscitation using the same neurologic deficit scoring system as developed for rats [15]. Similar to the results in rats [8], the neurologic outcome is dichotomous: mice were either dead soon after resuscitation, or survived with the appearance of nearly normal neurologic deficit scores on Day 3 and Day 10 after resuscitation.

**Figure 4.**
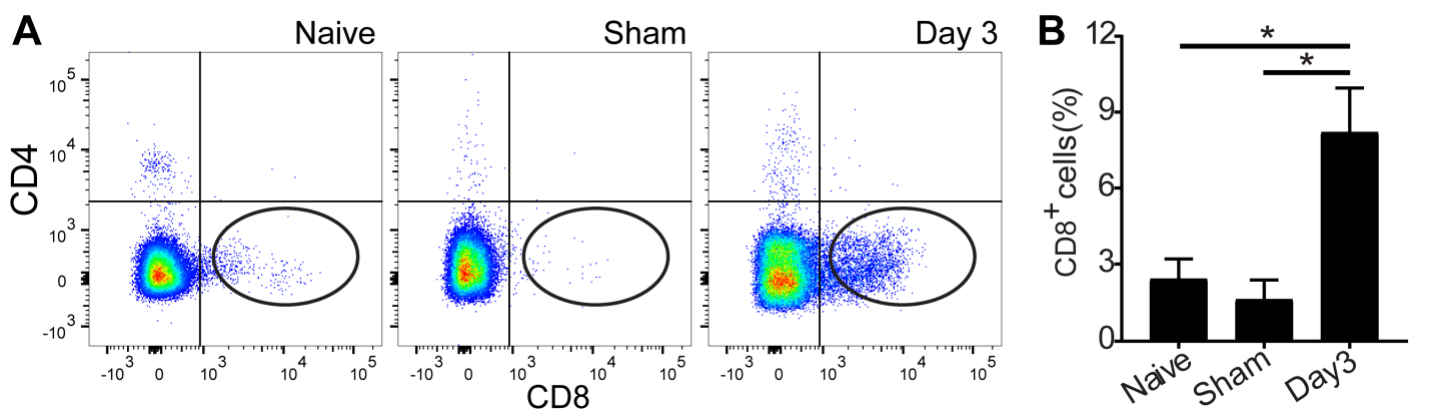
Infiltration of lymphocytes into the brain after cardiac arrest and resuscitation. (A) Flow cytometry evaluation of regulatory (CD4^+^) and cytotoxic (CD8^+^) T cell infiltration into the brain parenchyma 3 days after cardiac arrest and resuscitation, as compared to the naïve and time-matched sham controls. (B) Quantification shows nearly threefold increase in the CD8^+^T cell population in the brain tissue. Data are presented as mean ± SEM and analyzed using one-way ANOVA with least significant difference *post hoc* comparisons. *P < 0.05.

### Neuroinflammatory activation

Gliosis is a reactive response of the resident glial cells to various damages in the central nervous system (CNS), including ischemic damage [16, 17]. We quantified the hypertrophy of astrocytes and microglia in the CA1 region of the hippocampus by immunohistostaining for GFAP (Fig. 2A-2H) and Iba1 (Fig. 2I-2P), respectively. Three days after cardiac arrest and resuscitation, the percentage of pixels with GFAP^+^ immunoreactivity, determined by image segmentation and calculated against the total number of pixels in the region of interest, increased nearly 3-fold compared to the naïve mice (Fig. 2Q). A partial recovery in immunoreactivity of GFAP^+^ astrocytes was observed on Day 10 after cardiac arrest and resuscitation, albeit the level of GFAP^+^ immunoreactivity was still higher than that of the naïve and sham controls (Fig. 2Q). Similarly, on Day 3, the percentage areas of Iba1^+^ immunoreactivity increased nearly 4-fold in the cardiac arrest and resuscitation group, as compared to the naïve and sham controls. Notably, the level of Iba-1^+^ immunoreactivity on Day 10 returned to levels comparable to the time-matched sham controls (Fig. 2R). Hyperplasia and hypertrophy of astrocytes are clearly visible in the morphology of the GFAP^+^ cells 3 days after cardiac arrest and resuscitation (compare Fig. 2G to Fig. 2E and 2F). This reactive astrocytosis did not completely resolve 10 days after reperfusion (Fig. 2H). The morphological changes of Iba1^+^ cells followed the same trend as the astrocytes initially. On Day 3 after cardiac arrest and resuscitation, Iba1^+^ cells were mostly amoeboid, suggesting that they had past the primed stage and became macrophagic [18]. Unlike GFAP^+^ cells, however, Iba1^+^ microglia/macrophages returned to the ramified morphology after 10 days of recovery (Fig. 2P).

### BBB interruption

We assessed the BBB integrity by the variance from two T_1_-weighted MRI images acquired before and after the clearance of a blood-pool contrast agent. Representative T_2_- and T_1_-weighted images, T_1_ difference images, and variance maps of T_1_ difference images are shown in Fig. 3A. Unlike in focal ischemia, T_1_- and T_2_-weighted images are relatively insensitive to brain damage in global ischemia, but they provide accurate anatomic references to define regions of interest in quantifications. The images in each row in Figure 3A are displayed using the same intensity scale, so that the relative intensities over time can be compared. Hypointensity in the difference images at high magnetic field strength (14.1 T) suggests cancellation of relaxation enhancement due to trapping of the contrast agent into the brain tissue space outside the blood pool. As shown in Figure 3A, the BBB integrity was clearly compromised on the day of, and one day after, cardiac arrest. To determine the BBB damage on a microscopic scale, we calculated the pixel-by-pixel variance, as shown in the fourth row in Fig. 3A. Disseminated BBB leakages are clearly visible. The hippocampal regions of each animal are identified in the variance maps by co-registration with the corresponding T_2_-weighted images. Fig. 3B shows the mean pixel variances averaged for all hippocampal pixels of all animals as a function of time before cardiac arrest and after resuscitation, showing the time dependence of BBB leakages. BBB repair was observed starting 2 days after reperfusion.

**Figure 5.**
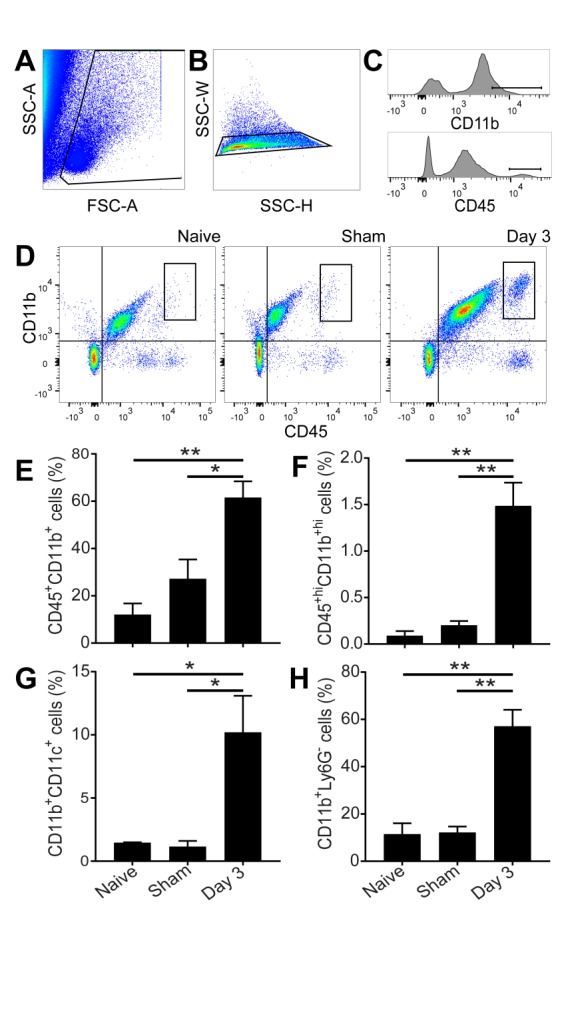
Infiltration of peripheral dendritic cells, monocytes, and macrophages into the brain parenchyma after cardiac arrest and resuscitation. (A-C) Gating strategies to isolate infiltrating immunocytes. (D) Representative flow cytometry data showing distinct CD11b^+hi^CD45^+hi^ cell population (boxes) in the brain 3 days after cardiac arrest and resuscitation. (E) Quantification shows a twofold increase in the CD11b^+^CD45^+^ after cardiac arrest and resuscitation. (F) Among the CD11b and CD45 doubly positive cells, the subpopulation of CD11b^+hi^CD45^+hi^ increased more than six-folds. This subpopulation is likely of peripheral origin. (G and H) CD11b^+^CD11c^+^ dendritic cells and CD11b^+^Ly6G^-^ monocytes are also significantly increased in the brain 3 days after cardiac arrest and resuscitation. Data are presented as mean ± SEM and analyzed using one-way ANOVA with least significant difference *post hoc* comparison. **P < 0.01 and *P < 0.05.

**Figure 6.**
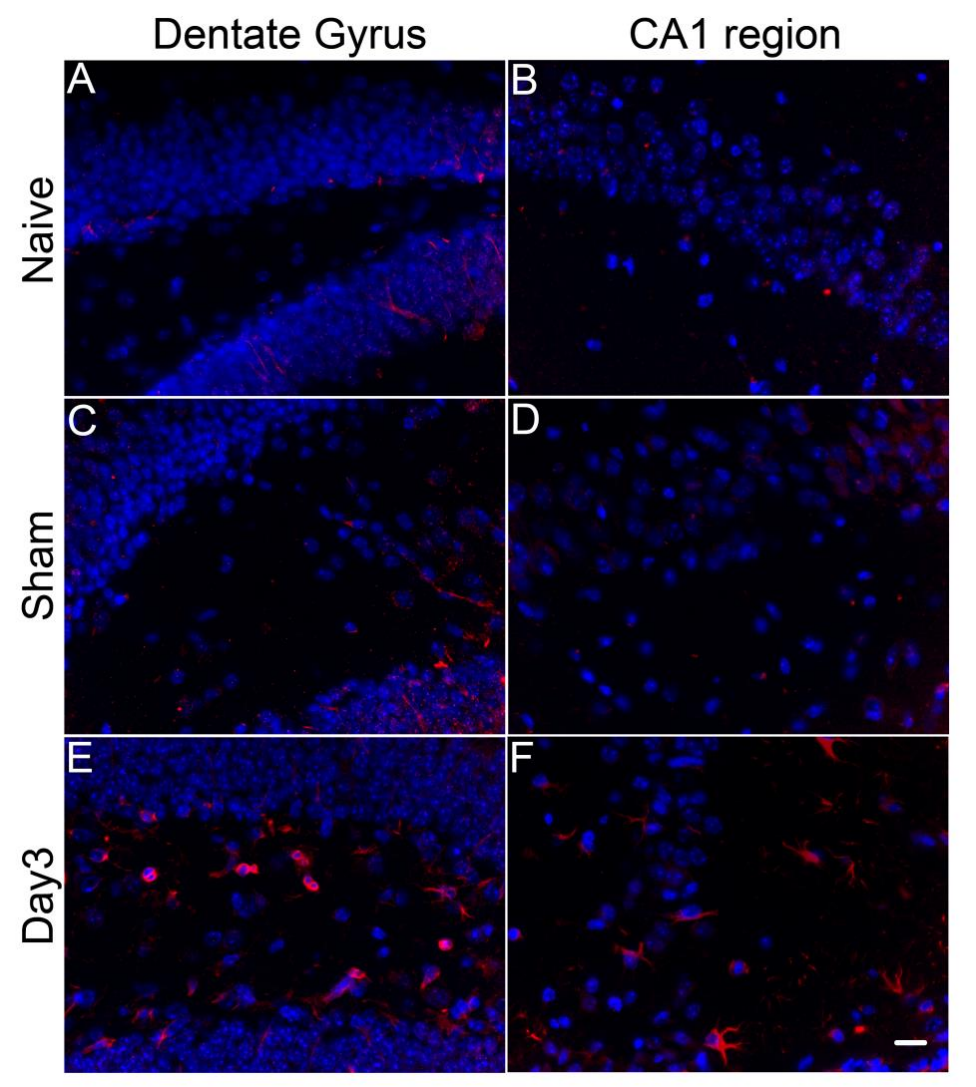
Spatial distribution of CD45+ cells in brain parenchyma. Immunohistochemical staining reveals that major changes in CD45^+^ immunoreactivity (red) occur in the dentate gyrus (A, C, E) and hippocampal CA1 region (B, D, F). Cell nuclei are co-stained by DAPI (blue). In naïve (A and B) and sham operated (C and D) mice, strongly CD45 positive cells are scarce. On Day 3 after cardiac arrest and resuscitation, there is a significant increase in the number of CD45^+^ cells in dentate gyrus and CA1 region. The strongly positive immmunoreactivity in E-F as compared to that in A-D is likely related to the CD45^+hi^ subpopulation in Figure 5. (Scale bar = 15 μm).

### Infiltration of CD8^+^ T cells into the brain

To determine the infiltration of immunocytes in response to neuroinflammation (Fig. 2Q and 2R), we performed flow cytometry on Day 3 after cardiac arrest and resuscitation to correlate immune cell infiltration with neuronal injuries. Detailed flow cytometry analyses of the brain tissue, which was perfused to remove blood cells from the microvasculature, revealed an increase in CD8^+^ T cells in the brain (Fig. 4A). On Day 3 after cardiac arrest and resuscitation, CD8^+^ T cells increased almost 3-fold in the brain (Fig. 4B). No difference in CD3^+^ and CD4^+^ T-cells was found between the sham controls and the mice undergone cardiac arrest and resuscitation (Fig. 4A). These results indicate a possible role of cytotoxic CD8^+^ T cells in peripheral immune response to CNS injuries.

### Infiltration of phagocytic leukocytes into the brain

To assess the role of monocytes/macrophages and dendritic cells in immune response to global ischemia, we analyzed the subset of phagocytic immune cells in the brain parenchyma. We first excluded cell debris based on forward scatter (FSC) by side scatter (SSC, Fig. 5A), followed by selection of single cell populations using SSC-H x SSC-W (Fig. 5B), and finally gated with antibodies for CD45, CD11b, CD11c, or Ly6G (Fig. 5C). The CD11b^+^CD45^+hi^ cells are gated as the double-positive subpopulations in the boxed region as shown in Fig. 5D. Similar gating strategies were used for other cell markers. We found that CD11b^+^CD45^+^ cell population significantly increased 3 days after cardiac arrest and resuscitation when compared to the naïve and sham controls (Fig. 5E). Most strikingly, CD11b^+^CD45^+hi^ cells, a subpopulation of microglia/macrophages, were distinctly present in the brain of the cardiac arrest group 3 days after resuscitation. This population was scarce in the controls and increased almost 6-fold after cardiac arrest and resuscitation (Fig. 5F). Similarly, CD11b^+^CD11c^+^ dendritic cells and CD11b^+^Ly6G^-^ inflammatory monocytes were also significantly increased in the brain tissue following cardiac arrest and resuscitation (Fig. 5G and 5H).

Although immunohistochemical staining is not a suitable quantitative method to differentiate various subpopulations of cells based on fluorescence intensities, staining with cell surface markers can nevertheless provide information about spatial distribution of invading cells. Fig. 6 shows CD45 staining of animals in the naïve, sham, and cardiac arrest groups. Most of the strong CD45^+^ immunoreactivity, possibly related to the invading CD45^+hi^ population, can be found in the dentate gyrus and the CA1 region of the hippocampus. Some, but not all, of these cells show the amoeboid morphology, characteristic of macrophage phenotypes.

**Figure 7.**
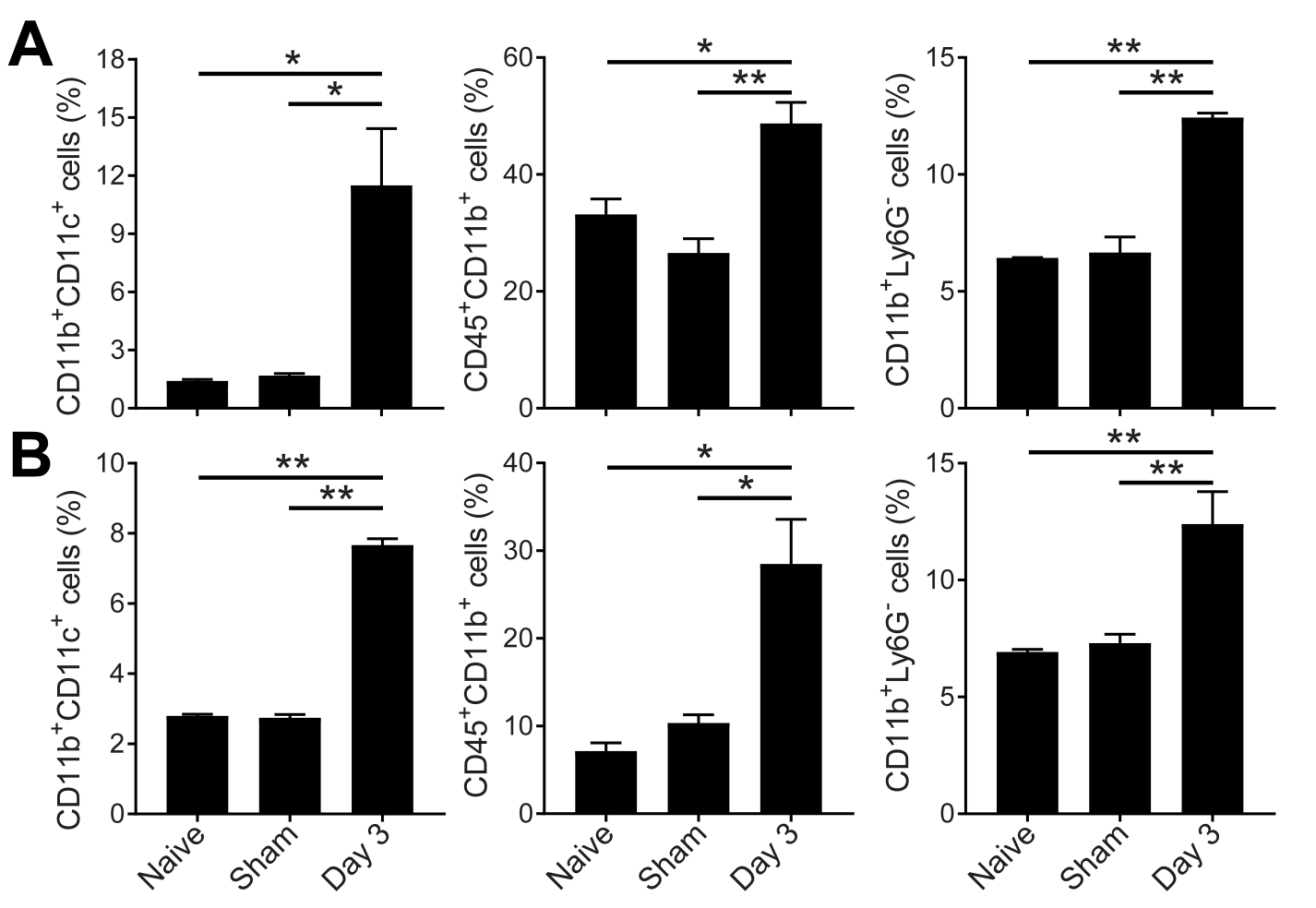
Flow cytometry of peripheral immune response to cardiac arrest and resuscitation. Quantitative evaluation of peripheral dendritic cells, monocytes, and macrophages in the bone marrow (A) and the blood (B). Data are presented as mean ± SEM and analyzed using one-way ANOVA with least significant difference *post hoc* comparison. **P < 0.01 and *P < 0.05.

### Peripheral immune response to global ischemia

As expected, peripheral immune cell populations changed significantly after global ischemia. In the bone marrow and blood, the CD11b^+^CD11c^+^ dendritic cells greatly increased 3 days after cardiac arrest and resuscitation. To a lesser degree, CD11b^+^CD45^+^ and CD11b^+^Ly6G^-^ monocytes and macrophages were also elevated (Fig. 7).

## DISCUSSION

We present here a new cardiac arrest and resuscitation model in mice. Our model is different from the previous KCl-overdose models in that it is highly reproducible and clinically relevant, without the need for chest compressions at an unmanageable rate of 5 compressions per second continuously for up to 120 s [13]. The esmolol dose used in our model is comparable to that used in heart transplantations in humans. The resuscitation by oxygenated blood infusion mimics the clinical setup of cardiopulmonary support. We showed that this model can produce characteristic ischemic injuries to the selectively vulnerable neurons in the CA1 region of the hippocampus. The spatial injury pattern resembles what has been reported previously in rats [8, 9] and mice [13]. However, the temporal profile of injury severity in mice differs from that in rats. Specifically, when the injuries are measured by the unhealthy neurons in the hippocampal CA1 region using conventional H&E staining, an apparent histological recovery was observed on Day 10 as compared to Day 3 after cardiac arrest and resuscitation. This finding is rather unexpected and challenges the traditional viewpoint that neuronal injury is irreversible after global ischemia. Although some very minor artifacts from the automatic tissue stainer are visible in the H&E sections, the neuron counting by 5 investigators, who were blinded to tissue identities, all showed a decrease in the number of unhealthy pyramidal neurons in the hippocampal CA1 regions on Day 10 relative to Day 3. A similar recovery based on CA1 neuronal counting was also reported in a different cardiac arrest model using neonatal mice [13]. In that study, the recovery was attributed to the disappearance of dead neurons on the longer recovery time, leading to an apparent decrease in the percentage of damaged neurons among the remaining neurons observable by histology. In our studies using adult mice, the histology damage, when measured by the unhealthy neurons (as opposed to dead neurons), seems to be more severe on Day 3 than on Day 10. Among many possible interpretations, the simplest is that the unhealthy neurons on Day 3 might have, in part, recovered by Day 10. Strongly associated with the reduction of unhealthy neurons on Day 10 is the recovery of neuroinflammation as measured by the activation of astrocytes and microglia. The role of microglia in various models of neuronal injuries has been extensively studied [19]. In global ischemia, the morphological changes from ramified to amoeboid shape, as seen in Figures 2M-2P, indicates a transition from an ATP-dependent activation to a hypoxic or anaerobic energy metabolic state with concomitant switching from activated microglia to macrophage function [19]. Using a similar cardiac arrest and resuscitation model in rats and non-invasive MRI cerebral perfusion measurements, we showed previously that cerebral perfusion was severely reduced within hours after resuscitation, and this hypoperfusion was independent of cardiac output and could last up to 48 hours [9, 10]. The transient anoxic condition during circulatory arrest and the protracted hypoxic condition during the critical phase of reperfusion can trigger environment-dependent extracellular signaling through the production of inflammatory cytokines and the commitment of pro-inflammatory and anti-inflammatory polarization of microglia and macrophages. The parallel time dependence of neuronal recovery and the resolution of neuroinflammatory changes in reactive microglia and astrocytes from Day 3 to Day 10 (compare Fig. 1 and Fig. 2) implicate an inflammation-related neuronal injury and repair process.

In addition to the innate immune reaction by the resident immune cells in the CNS, infiltration of peripheral immune cells in response to the ischemic insults also plays a role in shaping the progression of neuronal injury and repair. While conventional T_1_- and T_2_-weighted images provide a high-resolution anatomic reference for imaging analyses, they are usually less sensitive to changes in global ischemia (see top two rows in Fig. 3A). In contrast, the processed MRI variance maps from the difference of two T_1_-weighted images before and after contrast washout (Fig. 3) are able to reveal robust changes in pixel-by-pixel variances immediately after resuscitation and one day after reperfusion. These changes are a direct measure of the deterioration of the BBB integrity. The MRI variance images suggest that the global ischemic event during no-flow caused transient disruption to the BBB. The leakage was not fully repaired until two days later. During this period, blood-borne immune cells, including leukocytes, monocytes, macrophages, and the antigen-presenting dendritic cells, were all likely to gain access to the brain parenchyma. Although the time courses of the BBB disruption and neuronal injuries are not expected to match, it is conceivable that the BBB leakage can trigger neuroinflammatary responses, inducing immunocyte infiltration into the brain and setting the stage for subsequent neuronal damage. Indeed, cytotoxic T cells were found in high abundance in the brain 3 days after cardiac arrest and resuscitation (Fig. 4). These cytotoxic killer cells undoubtedly contributed to the maturation of neuronal injuries following the ischemic event. It should be noted that unlike CD8^+^ cells, the CD4^+^ regulatory T cells in the brain did not increase more than the sham controls in this study (Fig. 4A). This finding differs from a previous report in C57BL/6 and C57BL/6J mice, in which both CD4^+^ and CD8^+^ T cells were found to infiltrate into the brain several hours after cardiac arrest and resuscitation [20]. The discrepancy between the current study and the previous report warrants further investigations. Possible contributing factors include differences in animal strains, the time points at which brain tissues are collected, or both. However, the preference of the CD8^+^ T cells over CD4^+^ T cells to infiltrate across the BBB, as observed in this study, is consistent with other pro-inflammatory neuroimmune diseases [21] as well as the results in permanent focal ischemia models [6, 22]. CD8^+^ T cells have been found to be responsible for the dysregulation of the BBB through activation of astrocytes and alterations to the cerebral endothelial tight junction proteins in a perforin-dependent manner [23]. CD8^+^ T cells are also implicated in the initiation of neuronal expression of vascular endothelial growth factor, which is correlated with the regulation of the BBB permeability [24].

The MRI variance map presented in Fig. 3 is a novel and robust technique for noninvasive detection of blood-brain barrier leakage. It has long been speculated that the BBB is compromised after cardiac arrest and resuscitation, and now we have an MRI method to quantify its time course. Importantly, the approach presented here is readily applicable to human scanners. This new technology advancement will lead to meaningful changes in future clinical practice for the diagnosis and treatment of aging-related neurological diseases [25].

The distinct sub-populations of the CD11b^+hi^ CD45^+hi^, CD11b^+^CD11c^+^, and CD11b^+^Ly6G^-^ macrophage lineages in the brain parenchyma (Fig. 5 and 6) are unambiguous evidence that peripheral myeloid-derived monocytes and dendritic cells have infiltrated into the brain parenchyma and contributed to the immune modulation of neuronal injury process after global cerebral ischemia. In various tissues, mature macrophages are activated as an efficient response to pathogens. Their incredible plasticity underlies the different polarization phenotypes to markedly alter both the innate and adaptive immune responses [26, 27]. Revealing how microglia and peripheral monocytes communicate or regulate each other in response to ischemic insults will help advance the understanding of the neuronal injury and recovery mechanisms. Microglia, which are brain-specific macrophages, can potentially respond to pathogens by clearing neurotoxins and by secreting and regenerating neuroprotective signals. Importantly, in both acute and chronic brain injuries, microglia activation can release inflammatory mediators such as cytokines [28-30]. These activated microglia recruit monocytes, resulting in lymph cell infiltration into the diseased sites. As revealed in our data, CD11b^+hi^CD45^+hi^, CD11b^+^CD11c^+^, and CD11b^+^Ly6G^-^ populations migrated into the injury area. The mechanisms linking the activation and infiltration of immunocytes to the initiation of neuronal injuries should be further investigated.

Monocyte-derived brain macrophages do not persist in the CNS after inflammation has subsided. It appears that the blood-borne microglia/macrophages do not contribute to the long-living microglia nor reverse to become resident microglia. The fact that monocytes are also significantly increased in the bone marrow and blood (Fig. 7) suggests the possibility that peripheral immune response and CNS immunity are strongly coupled during the development and final resolution of neuronal injuries after cardiac arrest and resuscitation. This coupling represents unique opportunities to develop novel immune-based therapies for neuronal protection and repair by programming the pro- and anti-inflammatory signals in the periphery. For example, by manipulating and controlling bone-marrow cell lineages, it is possible to time the systemic immune responses to initiate neuronal repair at critical stages of reperfusion. Because the peripheral monocytes and macrophages can cross the BBB after cardiac arrest, these cells can also serve as vehicles for targeted drug delivery into the brain. Moreover, establishing correlations between peripheral immune cell profiles and long-term neurological outcome after cardiac arrest and resuscitation will allow robust tests to be developed to predict the outcomes of cardiac arrest patients in a clinical setting.
